# Survey of Sachet Water Waste Disposal in Liberia

**DOI:** 10.5696/2156-9614-8.20.181211

**Published:** 2018-12-06

**Authors:** Chikamso C. Apeh

**Affiliations:** ECOWAS Volunteer Program, Monrovia, Liberia

**Keywords:** plastic waste, sachet water, environmental safety, waste recycling

## Abstract

**Background.:**

Five hundred milliliter bags of water, referred to as ‘sachet water,’ are widely used in Liberia, as they are low cost, safe sources of clean drinking water for the population.

**Objectives.:**

This study aims to determine sources of drinking water in the study area, the rate of sachet water use, empty sachet water disposal methods and environmental problems associated with sachet water waste disposal.

**Methods.:**

Using a simple random sampling technique, 257 respondents were interviewed between April and June 2018 using structured questionnaires. On a five-point Likert scale with a mean score of 3 as the cutoff point, data were analyzed using descriptive statistics.

**Results.:**

The paper found that sachet water (mean (M)=4.37) is an essential source of drinking water in Liberia with a daily consumption rate of at least 6 bags of sachet water per individual. Affordability, availability, and safety were named by respondents as reasons for their consumption of sachet water. Improper disposal methods practiced by the residents of Liberia included ground littering (M=3.42) and burning (M=3.03).

**Conclusions.:**

Sachet water consumption has contributed to environmental issues such as drainage system clogs, littering of the environment, the death of terrestrial and aquatic animals due to plastic waste consumption, reduction of oxygen for aquatic life and soil infertility. We recommend the creation of policies and enforcement of sachet water production to include reuse and recycling of sachet water by-products as a requirement for licensing of producers, provision of adequate waste bins and the use of public education campaigns to educate residents on environmental best practices.

**Ethics Approval.:**

This study was approved by the Institute for Population Studies, University of Liberia, Monrovia, Liberia.

**Informed Consent.:**

Obtained

**Competing Interests.:**

The authors declare no competing financial interests.

## Introduction

Sachet water is a package of five hundred milliliters of water in a non-biodegradable synthetic polyethylene (polythene) mini-bag.^1^ It is widely known as “pure water” and its consumption is growing in developing countries, including Liberia, due to population increase and socioeconomic factors.^2^ It has become the primary source of drinking water for urban dwellers because it is affordable and accessible.^3^ The quantity of polythene mini-bags from pure water littering streets, farms and water bodies reflects its high level of use. Polythene bags can remain intact for 1000 years in the environment before they decompose and around 500 billion plastic bags are used every year worldwide.^4^,^5^

Sachet water is sourced directly from a pipe or borehole or a storage tank. It undergoes an industrial filtration process, sealed in sachets or plastic bags, and consumers believe it is safe after it is approved by the Ministry of Health to be hygienically safe for consumption. However, the quality of these sachets water in Liberia has yet to be studied.

Uncontrolled disposal of waste bags has caused several environmental problems such as land pollution (non-degradability of polythene in the soil), air pollution (burning of polythene), water pollution (underground water contamination), and sewage and drain blockage (flooding).^6^

Sachet water is produced and sold in shops, on the street, or delivered to homes. Statistics on sales of 500 ml sachet water are difficult to obtain, although consumption is increasing rapidly, especially in developing countries.^7^ Assuming the average daily consumption of water per person is six packs (3000 ml), this generates six empty sachets of waste per person per day. Multiplied by Liberia's population of 4,845,075, this means that approximately 29.1 million empty sachets water bags are generated daily.^8^ Empty sachet water bags are improperly disposed of and litter the environment. Some are not segregated before disposal and are found outside of house premises, streets, markets, churches, schools or any common area garbage dump within a locality, mixed with other decomposable and non-decomposable waste items.

According to Liberia's Public Health Law of 1975, which is still in effect, city corporations are responsible for ensuring clean and sanitary environmental conditions in Liberia.^9^ According to this law, city corporations are responsible for sanitation maintenance, including the cleaning, collection, and disposal of generated solid waste. For instance, in Monrovia, the Monrovia City Corporation and the Liberia Water and Sewage Corporation oversee waste management and enforcement of environmental laws. Their services include the management of solid waste disposal sites and enforcement of environmental and sanitation laws and regulations, prohibition of waste littering and enforcement of regulations requiring residents to clean their premises up to the sidewalk. However, these corporations have not been able to discharge their mandate due to constraints such as finance and logistics. According to the United Nations Environment Programme, in terms of equipment, Monrovia City Corporation has only two functioning tipper trucks for waste collection in a city of over 1.3 million people.^9^ In addition, six vehicles remain dysfunctional due to minor mechanical faults. Due to inadequate funding, Monrovia City Corporation has been unable to purchase the required spare parts. Although some private waste management companies are beginning to provide waste management services, solid wastes, predominantly empty sachet water bags, remain a problem.

Poor waste management capacity and lack of awareness of the negative effects of polythene bags has led to serious environmental, human and animal health impacts.^10^,^11^ To the best of our knowledge, there have been no studies examining the environmental impact of sachet water waste disposal in Liberia. Unfortunately, research in Liberia in the area of environmental sustainability remains limited due to ongoing reconstruction following the war. The present study attempts to fill this gap in the literature by not only contributing to renewed interest and relevance of environmental research but also to provide survey data as the basis for future empirical studies. Thus, this study aims to determine sources of drinking water in the study area, the rate of sachet water use, empty sachet water disposal methods and environmental problems associated with sachet water waste disposal.

## Methods

The present study was conducted in Liberia, which is an independent West African state and Africa's oldest republic. It is bordered by Ivory Coast to the east, Guinea to the north, Sierra Leone to the west and the Atlantic Ocean to the south. It has a total surface area of 111,370 km^2^, consisting of 96,320 km^2^ of land and 15,049 km^2^ of water. Its north-south extent is about 465 km and its Atlantic Ocean coastline is about 520 km long.^12^ It has an estimated population of 4.8 million people (4,849,545), of which 50.1% is urban and 48.9% rural, with an average household size of 4.3 people.^8^,^13^ It is divided into 15 counties which are further subdivided into about 136 districts and clans.^14^

Data were collected from April to June 2018 using a structured questionnaire (*Supplemental Material*). A draft questionnaire was validated by two experts; a resource and environmental management expert and an agricultural economist from the Ministry of Agriculture, Liberia. A simple convenience random sampling technique was used to select 300 respondents from Monrovia, the capital city of Liberia. Limited funding was the major determinant of the number and location of respondents surveyed. Three hundred copies of the questionnaire were administered, but two hundred and fifty-seven (257) copies were properly filled out and returned and used for analysis, for a response rate of 85.6%. Respondents gave their consent by signing the consent form attached to the questionnaire prior to answering the questions in the survey. The study was approved by the Institute for Population Studies, University of Liberia, Monrovia, Liberia.

Survey methods differ from empirical methods and have some limitations; causal inferences, for example, cannot be drawn between variables. However, according to Blinder, surveys provide valuable facts that test and/or compliment empirical studies.^15^ Moreover, another possible concern about surveys is that respondents may deliberately hide accurate information and provide inaccurate responses.^16^ However, in the present survey, respondents were given no incentives and were not primed to provide certain responses. In addition, subject were only recruited from Monrovia, not country wide due to funding limitations. Therefore, the results cannot be generalized to a larger population. Further studies should involve more respondents from the larger 15 county area.

Data were coded and analyzed using the Statistical Package for the Social Sciences (SPSS) software. Descriptive statistics and a five-point Likert scale were used with responses scored as ‘Strongly agree’ (5), ‘Agree’ (4), ‘Undecided’ (3), ‘Disagree’ (2), and ‘Strongly disagree’ (1). The sum of the ratings was 15 points, which was divided by 5 to get a mean score of 3 as the cutoff point. Any responses higher or equal to 3 were categorized as ‘Agreed’, while those lower than 3 were categorized as ‘Disagree’. Respondents were asked about items such as the use of sachet water/pure water in the study area, method of sachet water waste disposal and the environmental problems associated with sachet water waste disposal.

## Results

The demographic characteristics in Table 1 show that the average age of the 257 respondents was 37 years, and 58.4% were female and 41.6% were male. The majority were single (37%), closely followed by married (26.1%). Twenty-eight percent (28%) had no formal education, some had attended high school (27.6%), followed by elementary school (26.1%). The majority (28.4%) work as business owners, followed by students (20.2%) and farmers (19.1%).

**Table 1:**
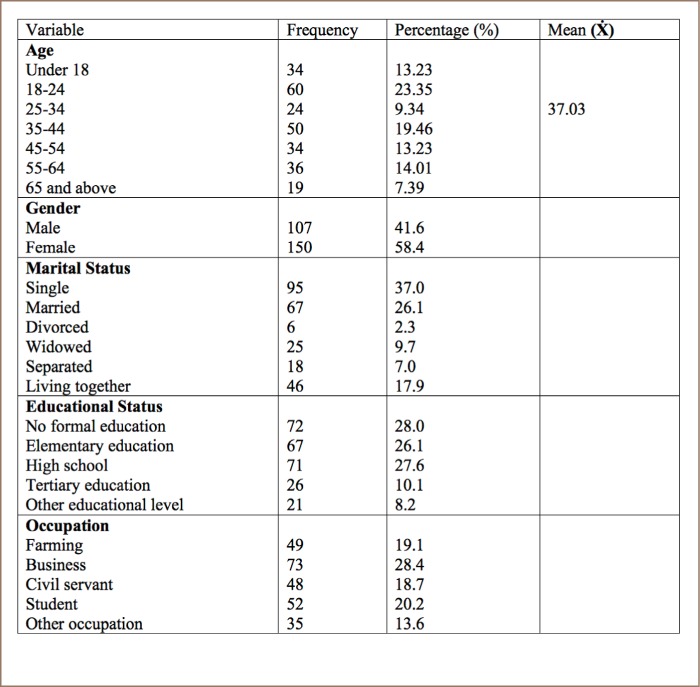
Demographic Characteristics of Respondents

The mean scores of respondents' sources of drinking water (*Table 2*) show that pipe or pump (mean (M)=3.65) and sachet water (M=4.37) are the two main sources of drinking water in the study area.

**Table 2:**
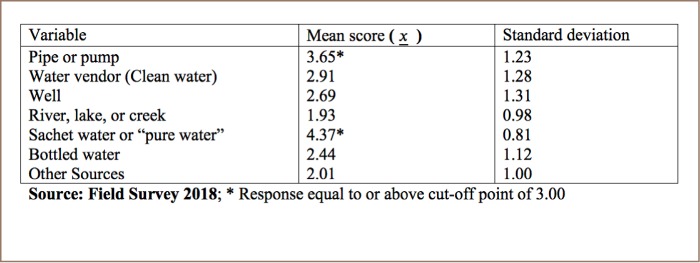
Reported Sources of Drinking Water

The results of the study in Table 3 reveal that all the respondents use sachet water, and their average daily consumption was 6 bags, which is equivalent to 3 liters of water (6 × 500 = 3000 ml).

**Table 3:**
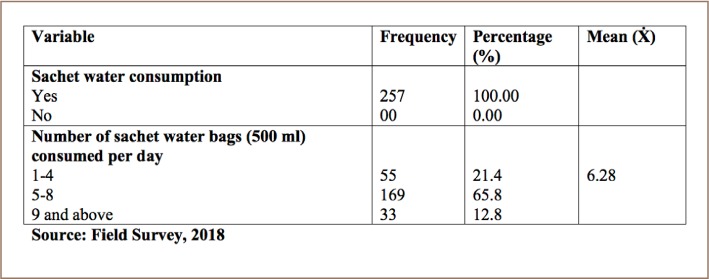
Rate of Sachet Water Use

Table 4 shows that the respondents gave the following reasons for consuming sachet water; affordability (M=3.19), safety (M=3.14) and availability (M=3.94).

**Table 4:**
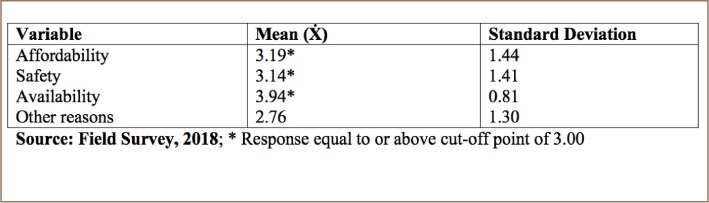
Reasons for Consumption of Sachet Water

The mean score of the respondents' sachet water waste disposal methods (Table 5) shows that waste bin (M=3.65), ground littering (M=3.42) and burning (M=3.36) were the most commonly used methods of sachet water waste disposal in the study area.

**Table 5:**
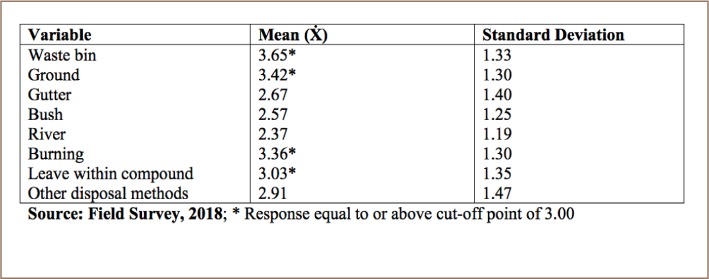
Sachet Water Waste Disposal Method

Table 6 shows that respondents are aware of the following environmental problems associated with improper sachet water waste disposal; block drainage systems, litter the environment, cause an offensive odor when burnt, breed mosquitoes, block the digestive tracts of ruminants after accidental ingestion, cause soil infertility when buried, pollute water bodies and detract from the natural beauty of the environment.

**Table 6:**
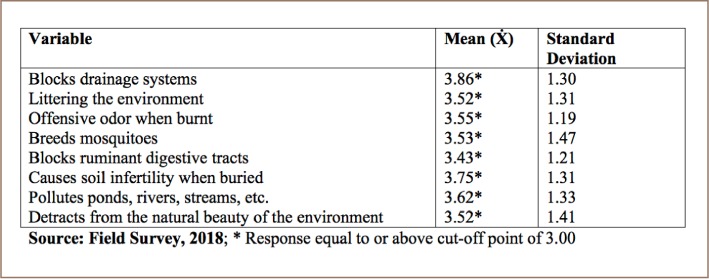
Environmental Problems Associated with Sachet Water Waste Disposal

## Discussion

Among the 257 respondents, 58% were female and 42% were male, with a mean age of 37 years. Most respondents were single (37%) with a poor educational background.

Only 10% had completed a tertiary education and 28% reported having no education due to factors such as poverty, long distances between homes and schools and poor infrastructure. Low levels of education were also found in a study in Liberia by the International Labour Office which found that economic reasons such as the inability to afford school fees or the need to earn an income were the main reasons reported by Liberians for not attending school.^17^ In addition, among those who did go to school, 26% left before graduation and two-thirds of early drop-outs cited the same economic reasons as their reasons for leaving school. Furthermore, the study found that 28.4% of the respondents worked as business owners to earn an income.

The study found that in Monrovia, most residents reported getting their drinking water from a pipe or pump and sachet water. Sachet water was the most predominant water source with a higher mean score (mean Likert score of 4.37) compared to pipe or pump water (mean Likert score of 3.65). Sachet water is the biggest source of drinking water in Monrovia as the public drinking water supply is unreliable. Pump water plays a supplementary role as households also have access to in-house pumps or a piped water supply. All respondents reported drinking sachet water and their average daily consumption was 6 bags, which is equivalent to three liters of water (6 x 500 = 3000 ml). This indicates that Liberians meet the conventional daily water intake of 3.7 liters for adult men and 2.7 liters for adult women.^18^ However, reliance on sachet water for daily drinking water needs has environmental consequences. The most important environmental concern for sachet water is the accumulation of plastic waste. Given the estimated population of 4.8 million people and consumption of 6 bags of sachet water per person daily, an estimated 29 million empty bags of sachet water waste are generated daily.

Consumption of sachet water is common among all income groups for a number of reasons, including affordability, safety, and availability. In a report by Miner et al., out of 360 respondents, 68% affirmed that sachet water is safe.^19^ A study by Omalu et al. stated that sachet water is a common source of drinking water in urban areas due to its availability and affordability.^20^ Olaniyan et al. found that reported reasons for sachet water intake are that it is cheaper, hygienic, pure and is considered safe.^21^

Sachet water waste disposal is a large problem because of its non-biodegradable nature, with serious implications for the environment. Sachet water bag waste can compromise soil fertility, contributing to food insecurity.^22^ Among the sachet water waste disposal methods in the present study, disposal in waste bins, ground littering and burning of waste were the most common methods of sachet water waste disposal reported by respondents.

Sachet water bag disposal methods coupled with poor drainage systems has led to several environmental issues. Disposal of sachet water wastes clogs drainage channels and prevents the free flow of water, causing gutters to overflow, thereby causing flooding, erosion and waterborne diseases. For instance, whenever rain falls, the street is flooded with floating sachet water waste. This improper sachet waste disposal causes water stagnation and contributes to the breeding of mosquitoes and an offensive odor in the major streets and slums of Monrovia city.

## Conclusions

The present study examined the environmental problems associated with sachet water waste disposal in Liberia. The findings show that sachet water is an important source of drinking water in Monrovia with a daily consumption rate of around 6 bags of sachet water per individual. There is a growing demand for sachet water due to its affordability, availability, and safety, but this demand has led to increased consumption and a glut of plastic waste, causing serious land, air and water pollution, which has adverse economic effects on the country. Residents use waste bins for empty sachet water disposal, but respondents also reported using methods such as ground littering and burning of waste. Finally, the study found that the inappropriate disposal methods practiced by the respondents have contributed to drainage system clogs, detract from the natural beauty of the environment due to littering, leads to the death of terrestrial and aquatic animals from accidental waste consumption, reduced oxygen levels in water bodies and loss of agricultural productivity. These consequences have not been given the needed attention, leading to continued improper waste disposal by residents across the city and beyond.

We recommend that safe drinking water be supplied to all parts of Liberia to reduce the use of sachet water to avoid environmental problems associated with it and the creation of effective policies and enforcement of sachet water production, including reuse and recycling of by-products as part of the requirement for licensing of producers to keep the city free from plastic wastes. Policymakers (government agencies and development partners) should mount waste bins at strategic positions for easy access to waste disposal.

Public awareness campaigns to educate residents on the consequences of improper waste disposal methods and the benefits of adopting conventional means of plastic waste disposal are urgently needed. One way to instill a culture of proper waste disposal in the younger generation would be to create clubs in schools to help educate the population in sustainable environmental management techniques.

The present study examined sachet water bag disposal methods and associated environmental consequences, as well as the socioeconomic characteristics of sachet water users. However, the correlation and causal relationship between population socio-economic factors and waste disposal methods are unclear. Further studies are needed for a fuller understanding of the issue of sachet water disposal, such as the effect of income on water source selection and the influence of education level on sachet water plastic waste disposal methods.
