# Supplementary material for: Survey of Sachet Water Waste Disposal in Liberia
Source: J Health Pollut. 2018 Dec 6;8(20):181211. doi: 10.5696/2156-9614-8.20.181211 (PMC6285681; doi:10.5696/2156-9614-8.20.181211)
Supplement: Supplementary file 1 [file hapn-8-20-181211_s01.docx]

**Survey of Sachet Water Waste Disposal in Liberia**

**Supplemental Material**

**Questionnaire on**

**Sachet Water Waste Disposal in Liberia**

**NB: Please Tick ( ) in any of the Boxes Provided Below that Reflects your choice
of options.**

**Section A: Biodata**

1. Age: ..............................................................
2. Gender: Male ( ) Female ( )
3. Marital status:
4. Single ( )
5. Married ( )
6. Divorced ( )
7. Widowed ( )
8. Separated ( )
9. Living together ( )
10. Educational Status:
11. No formal education ( )
12. Primary School ( )
13. High school ( )
14. Tertiary education ( )
15. (e) others specify ........................
16. Occupation:
17. Farming ( )
18. Business ( )
19. Civil servant ( )
20. Student ( )
21. others specify .............................

**Section B: Sources of drinking water**

1. What are your sources of drinking water?

| Drinking water source | Strongly agree | Agree | Undecided | Disagree | Strongly disagree |
| --- | --- | --- | --- | --- | --- |
| Pipe or pump |  |  |  |  |  |
| Water Vendor (Clean Water) |  |  |  |  |  |
| Well |  |  |  |  |  |
| River, lake, or creek |  |  |  |  |  |
| Sachet water or “pure water” |  |  |  |  |  |
| Bottled water |  |  |  |  |  |
| Other sources |  |  |  |  |  |

**Section C: Rate of sachet water use**

1. Do you drink Sachet water or “pure water”? Yes___ No____
2. How many bags (500 ml) of pure water do you drink in a day? _________
3. Why do you prefer pure water to other sources of water?

| Variables | Strongly agree | Agree | Undecided | Disagree | Strongly disagree |
| --- | --- | --- | --- | --- | --- |
| It is affordable |  |  |  |  |  |
| Safe |  |  |  |  |  |
| Available |  |  |  |  |  |
| Other Reasons |  |  |  |  |  |

**Section D: Empty sachet water disposal method**

1. How do you dispose the empty bag of pure water after drinking it?

| **Empty sachet water bag disposal method** | Strongly agree | Agree | Undecided | Disagree | Strongly disagree |
| --- | --- | --- | --- | --- | --- |
| In the waste bin |  |  |  |  |  |
| On the ground |  |  |  |  |  |
| In the gutter |  |  |  |  |  |
| In the nearby bush |  |  |  |  |  |
| In the nearby river |  |  |  |  |  |
| By burning it |  |  |  |  |  |
| Within the compound |  |  |  |  |  |
| Other Method |  |  |  |  |  |

**Section E:** **Environmental problems associated with sachet water waste disposal**

1. What are your perceived environmental problems associated with pure water waste disposal?

| **Environmental problems** | Strongly agree | Agree | Undecided | Disagree | Strongly disagree |
| --- | --- | --- | --- | --- | --- |
| Blocking of drainage systems |  |  |  |  |  |
| Littering of the environment |  |  |  |  |  |
| Gives out offensive odor when burnt |  |  |  |  |  |
| Breeds mosquitoes |  |  |  |  |  |
| Blocks ruminant tracts when eaten |  |  |  |  |  |
| Causes soil infertility when buried |  |  |  |  |  |
| Pollutes ponds, rivers, streams |  |  |  |  |  |
| Litters the environment |  |  |  |  |  |
| Causes illnesses such as malaria, diarrhea, cholera, nausea, typhoid, dermatological problems etc. |  |  |  |  |  |
